# Transabdominal pre-peritoneal hernia repair: risk of operation for recurrence depends on choice of both mesh and fixation device. A study from the Danish Hernia Database

**DOI:** 10.1007/s10029-025-03344-5

**Published:** 2025-07-11

**Authors:** Alexander Mortensen, Anne Bodilsen, Hans Friis-Andersen

**Affiliations:** 1 Department of Surgery, Regionshospitalet Horsens, Horsens, Denmark; 2 Department of Surgery, Aarhus University Hospital, Aarhus N, Denmark

**Keywords:** Inguinal Hernia, Laparoscopic inguinal hernia repair, Mesh fixation, Risk of reoperation, Cohort study

## Abstract

**Purpose:**

Multiple methods of mesh fixation are available in laparoscopic inguinal hernia repair, as well as multiple types of mesh. No previous studies compare all methods of fixation in TAPP against each other in regards to risk of reoperation for recurrence. In addition, there is little data comparing types of mesh or the relationship between mesh and fixation method.

**Methods:**

We compare the tissue-penetrating methods with non-penetrative as well as no fixation, and examines the interaction of fixation method and choice of mesh. Cohort was established by way of the Danish Hernia Database, identifying patients operated with TAPP from Jan. 2010 to Dec. 2022. Cox’ regression analyses were performed, with multivariate analysis correcting for significant confounding variables, yielding adjusted hazard ratios (aHR) for reoperation for each fixation method. Follow-up analyses investigated whether differences in mesh types significantly impacted the results.

**Results:**

Among 49,029 TAPP repairs, 3.6% experienced reoperation for recurrence over a mean follow-up of 5.76 years. Tack fixation, the most common method, showed the highest reoperation rates (5.3% at 5 years). Glue, self-fixating meshes, and no fixation, had significantly lower risk in comparison (aHRs of 0.25, 0.21, and 0.51, respectively). Even after correcting for weight and pore size, some mesh types significantly impacted risk, with aHRs spanning 0.28 – 1.

**Conclusion:**

Non-penetrative fixation methods and no fixation are associated with lower reoperation rates compared to tissue-penetrative methods, with self-fixating meshes carrying the lowest risk. In addition, we found significant differences in aHR between types of mesh.

## Background

Inguinal hernias are common, and patients are often affected in regards to discomfort, pain, and limited physical activity. Although rare in inguinal hernias, cases with incarceration and strangulation may result in serious morbidity and even mortality. Inguinal hernia repair is among the most commonly performed surgical procedures worldwide [1–3]. It is considered a low-risk procedure with minimal short term adverse outcomes. In the long term, there is however a risk of developing chronic pain, sexual dysfunction, and recurrence [4–7]. Due to the high number of inguinal hernia repairs carried out every year, risk of recurrence remain an important measure for health outcomes, patient satisfaction, and socio-economic considerations.

The recommended treatment for inguinal hernia is mesh repair (synthetic, biological, or combined) [8], either with open or laparoscopic approach [8–10].

Mesh fixation technique in the laparoscopic transabdominal pre-peritoneal approach (TAPP) is largely dependent on surgeon or center preferences. Different methods of mesh fixation have been associated to chronic pain and hernia recurrence, although results vary especially in regards to efficacy of tack fixation compared to other methods [4, 6, 11–23]. Most studies compare outcomes in tissue-penetrative fixation methods versus non-penetrative fixation methods, or no fixation [11, 12, 14–16, 18–22, 24–31]. To our knowledge, no studies have investigated recurrence rates comparing all mesh fixation methods in a single study population.

Experimental studies show that different types of mesh have different reactions to implantation in regards to shrinkage and adhesion [32], and this reaction further varies with choice of fixation device [33]. A recent study supports this finding in a clinical setting [34]. Accordingly, it seems relevant to consider mesh type and fixation device in addition to classic factors leading to recurrence.

In the present study, we seek to investigate rates of reoperation for inguinal hernia recurrence in elective patients receiving TAPP repair, aiming to compare risk of reoperation for recurrence in no fixation versus both tissue-penetrative mesh fixation and non-penetrative mesh fixation on a thoroughly detailed patient population. We will also investigate whether choice of mesh is a significant factor in recurrence compared to choice of fixation method.

## Methods

### Participants and data acquirement

We obtained data from The Patient Safety Authority's Online Register and The Danish Hernia Database, a nationwide database prospectively recording detailed information on hernia operations in Denmark since 1998 with a data completeness of 95%. After any hernia repair, the surgeon reports perioperative data and patient-related factors in the database. Approximately 10,000 inguinal hernia operations per year are registered. A detailed description of methodology and limitations of the database is described elsewhere in detail [35].

The inclusion criteriae were age ≥ 18 years and primary elective operation for inguinal or femoral hernia using TAPP between January 2010 and December 2022.

Exclusion criteriae were open approach, perioperative conversion to alternative approach, such as Lichtenstein's procedure, robot-assisted or totally extraperitoneal repair, findings of alternative pathology such as tumor, undescended testicle, femoral artery aneurism etc., and signs of strangulation. Data entries with unclear or defective reporting were also excluded from analysis.

### Endpoints of interest

Primary endpoint was the reoperation rate and risk of reoperation due to recurrence after glue fixation of mesh, self-adhesive mesh fixation, no fixation, or tissue-penetrating fixation in the form of clips, sutures and tacks, indicated by 5-year reoperation rate and hazard ratio. Recurrences were defined by the findings of an ipsilateral reoperation following previous TAPP repair. For each patient, multiple recurrences in the same side were not counted separately.

Previous recommendations on choice of fixation method have depended on hernia size and medial hernia [8]. We included analyses on larger and smaller defects for each fixation method, as well as for medial and pantaloon hernias. In these analyses, hernia size or type were appropriately disincluded from multivariate correction.

Secondary endpoints were differences in risk of reoperation between the various mesh types utilised in Denmark during the study period, with a follow-up analysis of whether these differences significantly impact the results of the primary endpoint. The impact of density and pore size of mesh for risk of reoperation was also investigated, as was the impact of absorbable tacks versus non-absorbable.

### Statistical analysis

All statistical analyses were done using STATA (StataCorp LP, College Station, TX) with a threshold of significance of 5%.

Prior to analysis of the endpoints, potential confounding variables in patient characteristics were identified. Differences of continuous variables (age, hernia size, mesh size) were examined using student's t-test subsequent to testing for normal distribution and equal variance for continuous data by histograms and Q-Q plots. For data not following a normal distribution, medians were used instead of means with a non-parametric Mann–Whitney U test. To evaluate potential difference in reoperation dependent on center caseload, centres were pooled into groups depending on their annual procedural volume as noted by the Danish Hernia Database. Groups were as follows: low volume (1–99 cases/year), medium volume (100–249 cases/year) and high volume (exceeding 250 cases/year). Categorical characteristics (right/left side surgery, supervised procedure, sex, hernia specification, sliding hernia, center volume) were tested using χ^2^ test. Statistically significant differences in variables were identified and factored in subsequent multivariate analyses.

Cox proportional hazards regression analyses were used to assess the crude hazard ratios (HR) of reoperation relative to method of mesh fixation or lack thereof. Patient characteristics with significant associations were included for further analysis in a multivariate model correcting for the other independent variables, computing adjusted hazard ratios (aHR). To show the unadjusted cumulative reoperation risk by either fixation method or mesh use, Kaplan–Meier plots were used.

To investigate whether choice of mesh had a significant effect on risk of reoperation, we repeated cox regressions for meshes rather than fixation method, focusing on meshes with at least 5 years observation period and at least 50 includable patients for analysis feasibility. To analyse whether choice of mesh could significantly alter the risk of reoperation for a method of fixation, we repeated this analysis for each fixation group separately.

The largest subgroup was chosen as reference for all regression analyses.

Difference in use of absorbable or permanent tacks between the groups of reoperation or no reoperation was tested using χ^2^ test, and combinations of high or low weight meshes (± 50 g/m2) with small or large pore size (± 1 mm) was also compared using multivariate Cox proportional hazards regression analyses.

## Results

### Participation and patient characteristics

**Fig. 1:**
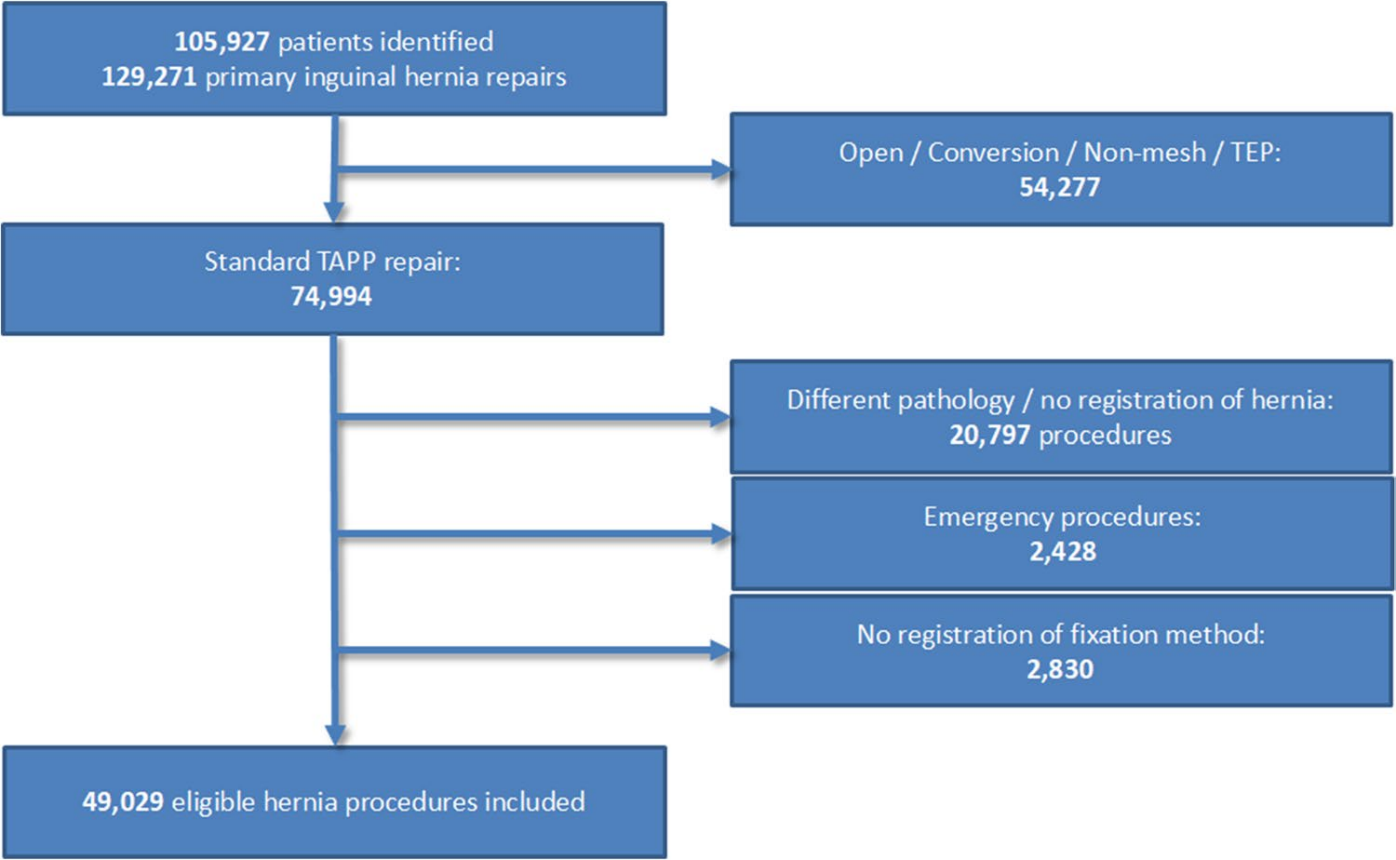
Flow-chart of patient inclusion

A total of 129,271 hernia repairs were identified during the trial period (156 months) as having received mesh implantation in relation to primary inguinal and femoral hernia repair. Of these, 49,029 had TAPP repair and was included in the study (Fig. 1). A total of 2,830 of the identified 129,271 groins (2.2%) had insufficient data for analysis.

Of the included 49,029 hernia repairs, a total of 1,752 had reoperation for recurrence during the follow-up (FU) period (3.6%). The mean FU was 2101 days, or 5.76 years. The most commonly used method of fixation was tacks (23,285, 47.5%), followed by glue fixation (9,181, 18.7%), then clips (6,167, 12.6%), self-fixation (5,075, 10.4%), no fixation (3,065, 6.25%), and lastly sutures (694, 1.4%).

Male sex, sliding, medial, and pantaloon hernias, hernia size and center volume were found to have statistically significant association to reoperation, and subsequent multivariable Cox’ regression analyses corrected for these variables. No correlation was found to mesh length, width or area, nor whether the operation was supervised (Table 1).

**Table 1:** Patient and peroperative characteristics

	Reoperation for recurrence	No known recurrence	*P*-value
	*n* = *1,752*	*n* = *47,277*	
Age at procedure, mean	56.54	56.92	*p = *0.293
Sex			***p* < *0.001***
Female	131 (1.6%)	7,910 (98.4%)	
Male	1,621 (3.9%)	39,367 (96.1%)	
Supervised operation			*p = *0.208
Yes	63 (2.1%)	2,941 (97.9%)	
No	695 (2.5%)	27,446 (97.5%)	
Hernia side			*p = *0.402
Right	954 (3.5%)	26,222 (96.5%)	
Left	798 (3.7%)	21,055 (96.4%)	
Hernia specification			***p* < *0.001***
Femoral	63 (2.6%)	2,386 (97.4%)	
Lateral	923 (3.4%)	26,112 (96.6%)	
Medial	635 (3.8%)	16,143 (96.2%)	
Pantaloon	117 (5.0%)	2,224 (95.0%)	
Sliding hernia			***p* < *0.001***
Yes	187 (5.7%)	3,112 (94.3%)	
No	908 (3.0%)	29,454 (97.0%)	
Avg. hernia size, fingers width	2.44	2.76	***p* < *0.001***
Mesh length (mean)	12.65 cm	12.69 cm	*p = *0.736
Mesh width (mean)	13.25 cm	13.44 cm	*P = *0.202
Mesh area	196.83 cm2	206.02 cm2	*P = *0.128
Center volume			
Low	589 (5.3%)	10,471 (94.7%)	***p* < *0.001***
Medium	200 (3.2%)	5,993 (96.8%)	
High	1001 (3.6%)	26,868 (96.4%)	

The data of this study are available on request from the corresponding author in anonymized form. The data are not publicly available due to concerns of participant privacy and restrictions of the utilized database

### Reoperation by method of fixation

**Fig. 2:**
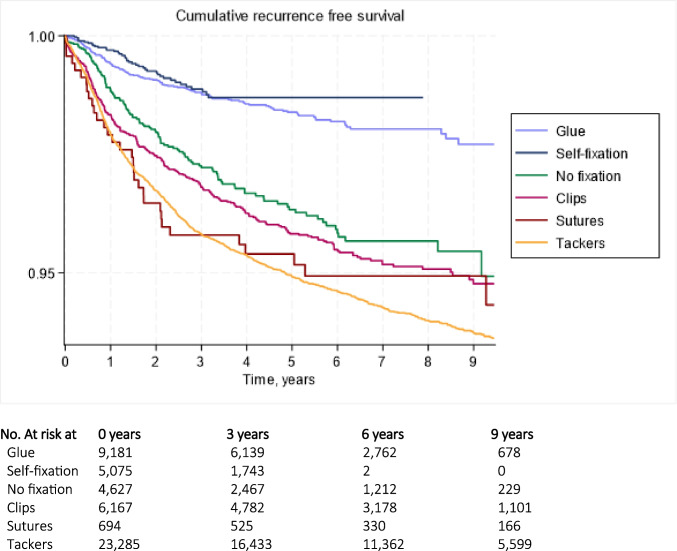
Kaplan–Meier plot showing reoperations by fixation method

The reoperation rates at 5 years FU varied from 1.3% to 5.2%, with self-fixation and glue fixation having the lowest rates and tack fixation having the highest (Table 2). As tack fixation was the largest group, it was chosen as reference for hazard analysis. The univariate Cox regression computed HRs ranged from 0.25 (self-fixating) to 0.98 (sutures) and 1 (tack fixation, reference value). The unadjusted cumulative reoperation free survival is depicted as a Kaplan–Meier plot in Fig. 2.

**Table 2:** Fixation method comparison

Mesh	No	5y Reoperation	HR	aHR (95%-CI)
Glue adhesive	9,181 (18.7%)	1.8%	0.32	***0.28 (0.23–0.38)***
Smaller defect^1^	5,299 (23.8%)	1.4%	0.34	***0.32 (0.23–0.43)***
Larger defect^2^	2,877 (17.3%)	2.1%	0.22	***0.19 (0.11–0.30)***
Medial Hernia	2,597 (15.5%)	2.1%	0.33	***0.15 (0.04–0.64)***
Combined Hernia	275 (11.8%)	2.8%	0.46	***0.33 (0.12–0.90)***
Self-fixation	5,075 (10.3%)	1.3%	0.25	***0.24 (0.18–0.32)***
Smaller defect	2,218 (9.9%)	1.4%	0.30	***0.24 (0.14–0.42)***
Larger defect	2,812 (16.9%)	*None*	0.20	***0.18 (0.11–0.31)***
Medial Hernia	1,688 (10.0%)	1.8%	0.26	***0.13 (0.03–0.54)***
Combined Hernia	285 (1.22%)	*None*	0.16	***0.16 (0.05–0.66)***
No fixation	4,627 (9.4%)	4.0%	0.71	***0.55 (0.39–0.71)***
Smaller defect	2,303 (10.3%)	3.5%	0.83	***0.70 (0.51–0.97)***
Larger defect	1,964 (11.8%)	5.9%	0.53	***0.43 (0.21–0.56)***
Medial Hernia	1,455 (8.7%)	5.4%	1.05	1.01 (0.44–2.29)
Combined Hernia	183 (7.8%)	2.6%	0.48	0.58 (0.23–1.45)
Clips	6,167 (12.6%)	4.1%	0.82	0.85 (0.68–1.21)
Smaller defect	3,459 (15.5%)	4.2%	0.99	1.00 (0.79–1.27)
Larger defect	1,178 (7.1%)	5.9%	0.76	0.64 (0.45–1.22)
Medial Hernia	2,327 (13.9%)	4.0%	0.83	1.30 (0.59–2.88)
Combined Hernia	240 (10.3%)	7.1%	0.96	1.01 (0.51–1.99)
Sutures	694 (1.4%)	5.2%	0.98	0.77 (0.36–1.55)
Smaller defect	332 (1.5%)	4.4%	1.1	0.58 (0.22–1.57)
Larger defect	139 (0.8%)	2.5%	0.68	0.90 (0.34–2.4)
Medial Hernia	174 (1.0%)	6.6%	1.13	1.71 (0.23–12.6)
Combined Hernia	26 (1.1%)	6.7%	0.62	0.84 (0.12–6.16)
Tacks	23,285 (47.5%)	5.3%	1 (ref)	1 (ref)
Smaller defect	8,683 (38.9%)	4.8%	-	-
Larger defect	7,680 (46.1%)	7.2%	-	-
Medial Hernia	8,537 (50.9%)	5.1%	-	-
Combined Hernia	1,331 (56.9%)	6.9%	-	-

1: Defect size of 2 fingers width or smaller

2: Defect size larger than 2 fingers width

In the following multivariate correction, the differences between groups remained and proved statistically significant (Table 2). Adjusting for sex, hernia specification, center volume, sliding hernias and size, only the suture-group (aHR = 0.77) and the clips group (aHR = 0.85) had no significant difference to the tack fixation group (*p = *0.356 and *p = *0.146, respectively). The non-penetrative fixation groups had the lowest adjusted hazards (aHR = 0.28 for glue-fixation and aHR = 0.24 for self-fixation); the no fixation group proved closer to non-penetrative fixation in quality albeit with a slightly higher association to reoperation still, with a stastistically significant aHR of 0.55 (*p* < *0.001*). Of note, the relative superiority of the no fixation method was not evident when looking only at medial or pantaloon hernias, with 5 year reoperation rates similar to tack fixation (5.4% and 5.1% for medial hernias, respectively).

### Differences between meshes

The 5-year reoperation rates between meshes ranged from 1.4% (TiMesh) to 13.6% (Physiomesh). The UltraPro mesh was most commonly used and selected as reference for regression.

Of the 20 included meshes, eight had significantly lower aHR compared to the most widely used reference mesh, with none significantly higher: Bard 3D Light, Cousin, Galmesh, Galmesh Light, Optilene, Parietene and UltraPro Advanced. The lowest aHRs with significant difference was ProGrip (aHR = 0.23), which is obligatorily self-fixating, and Optilene (aHR = 0.38).

When analyzing by mesh properties (weight and pore size), there was no significant differences in aHR.

The entire range of included meshes with 5-year reoperation, HR and aHR with 95%-CI are given in Table 3 and for mesh properties in Table 4.

**Table 3:** Mesh type comparison

Mesh	No	5y Reoperation	HR	aHR (95%-CI)
Bard 3D	708 (1.7%)	2.5%	0.50	1.06 (0.52–1.93)
Bard 3D Light	3,367 (8.1%)	2.8%	0.53	***0.59 (0.41–0.80)***
Bard Soft	2,473 (6.0%)	4.8%	0.92	0.76 (0.56–1.10)
Cousin	2,274 (5.5%)	3.5%	0.64	***0.62 (0.40–0.94)***
Galmesh	1,996 (4.8%)	3.4%	0.60	***0.56 (0.29–0.82)***
Galmesh Light	2,114 (5.1%)	4.0%	0.70	***0.65 (0.44–0.91)***
Klas	113 (0.3%)	10.9%	2.06	1.87 (0.75–5.11)
Optilene	3,015 (7.3%)	*None*	0.45	***0.38 (0.22–0.67)***
Optilene Elastic	310 (0.7%)	*None*	0.49	0.58 (0.23–1.51)
Parietene	6,676 (16.1%)	3.4%	0.59	***0.50 (0.40–0.64)***
Parietex	2,261 (5.5%)	5.3%	0.93	0.76 (0.51–1.07)
ProGrip	5,875 (14.2%)	2.7%	0.32	***0.23 (0.15–0.30)***
Physiomesh	53 (0.1%)	13.6%	2.21	0.83 (0.12–5.91)
Prolene	1,500 (3.6%)	5.3%	0.97	0.72 (0.39–1.07)
SupraMesh	314 (0.8%)	4.4%	0.75	0.70 (0.18–3.14)
SurgiPro	189 (0.5%)	8.8%	1.50	1.90 (0.79–4.16)
TiMesh	78 (0.2%)	1.4%	0.79	0.32 (0.04–2.23)
UltraPro	7,941 (19.1%)	5.1%	1 (ref)	1 (ref)
UltraPro Adv	1,502 (3.6%)	2.8%	0.52	***0.56 (0.36–0.76)***
VyPro	141 (0.3%)	8.3%	1.57	1.43 (0.75–2.49)

**Table 4:** Mesh density and pore size

Mesh	No	5y Reoperation	HR	aHR (95%-CI)
Large/Light^2^	35,741 (77.2%)	4.3%	1 (ref)	1 (ref)
Small/Light^1^	78 (0.2%)	1.4%	1.13	0.48 (0.07–3.38)
Large/Heavy^4^	4,004 (8.6%)	5.2%	1.25	1.15 (0.90–1.47)
Small/Heavy^3^	6,428 (13.9%)	4.6%	1.05	0.89 (0.69–1.15)

1: Mesh with small pore size and lightweight properties

2: Mesh with large pore size and lightweight properties

3: Mesh with small pore size and heavyweight properties

4: Mesh with large pore size and heavyweight properties

### Effect of mesh choice on risk of reoperation by method of fixation

For each fixation group, the risk of reoperation was evaluated based on choice of mesh. The most widely used meshes were the Parietene for the glue fixation group, Bard 3D Light for the no fixation group, Bard Soft for the clips fixation group, and UltraPro for the tack fixation group. These were set as reference for intragroup cox regression analysis. As the ProGrip was the only mesh in the self-fixation group, no analysis was done for this fixation method. For the suture fixation group, there was a complete match between reoperations and potential confounding variables, negating the cox regression and rendering the aHR uncomputable. Similar analyses by mesh properties were also carried out.

The data is shown in Tables 5, 6, 7, 8, and 9. No significant difference between meshes was found in the glue fixation group. In no fixation, the Cousin mesh had significantly higher hazard than the reference mesh, despite being among the better performing meshes in the analysis including all fixation methods. In tack fixation, seven meshes had significantly lower hazards than the reference (UltraPro); these were also significantly better in the analysis including all fixation methods.

**Table 5:** Glue fixation group

Mesh	No	HR	aHR (95%-CI)
Bard 3D	64 (0.9%)	1.00	2.70 (0.39–20.18)
Bard Soft	85 (1.2%)	3.19	2.97 (0.70–12.62)
Cousin	71 (1.0%)	0.92	1.69 (0.23–12.50)
Galmesh	123 (1.8%)	0.62	1.13 (0.15–8.38)
Galmesh Light	408 (5.8%)	0.52	0.48 (0.11–2.02)
Optilene	624 (8.9%)	1.04	1.35 (0.47–3.93)
Parietene	3,472 (49.4%)	1 (ref)	1 (ref)
Parietex	368 (5.2%)	1.46	1.16 (0.54–2.44)
UltraPro	1,418 (20.2%)	1.81	1.15 (0.55–2.44)
UltraPro Adv	359 (5.1%)	0.94	1.28 (0.49–3.35)
Versatex Monofil	1,043 (14.8%)	0.38	0.34 (0.08–1.47)
Large/Light	6,750 (78.1%)	1 (ref)	1 (ref)
Small/Light	3 (0.03%)	-	-
Large/Heavy	453 (5.2%)	0.96	0.93 (0.33–2.59)
Small/Heavy	1,437 (16.6%)	0.66	0.49 (0.18–1.38)

**Table 6:** No fixation group

Mesh	No	HR	aHR (95%-CI)
Bard 3D Light	624 (30.7%)	1 (ref)	1 (ref)
Bard 3D	58 (2.9%)	3.01	2.64 (0.54–12.82)
Bard Soft	470 (23.1%)	2.21	1.99 (0.74–5.33)
Cousin	491 (24.2%)	2.03	***2.81 (1.13–6.99)***
Galmesh Light	389 (19.1%)	1.10	0.72 (0.18–2.89)
Large/Light	3,600 (95.9%)	1(ref)	1(ref)
Small/Light	0	-	-
Large/Heavy	14 (0.37%)	-	-
Small/Heavy	139 (3.7%)	1.26	0.96 (0.23–3.96)

**Table 7:** Clips fixation group

Mesh	No	HR	aHR (95%-CI)
Bard 3D	255 (5.1%)	0.73	1.88 (0.78–4.57)
Bard 3D Light	924 (18.4%)	0.56	0.75 (0.34–1.67)
Bard Soft	1,432 (28.5%)	1 (ref)	1 (ref)
Cousin	573 (11.4%)	0.77	0.85 (0.45–1.63)
Galmesh Light	678 (13.5%)	1.39	1.44 (0.80–2.61)
Optilene	407 (8.1%)	0.98	0.89 (0.36–2.24)
Parietene	513 (10.2%)	1.18	1.28 (0.70–2.35)
Prolene	68 (1.4%)	0.26	0.53 (0.07–3.89)
UltraPro	566 (11.3%)	0.71	0.83 (0.36–1.89)
UltraPro Adv	205 (4.1%)	0.61	0.68 (0.23–1.94)
Large/Light	5,386 (88.8%)	1 (ref)	1 (ref)
Small/Light	0	-	-
Large/Heavy	133 (2.2%)	0.75	0.36 (0.05–2.58)
Small/Heavy	548 (9.0%)	0.88	0.96 (0.42–2.20)

**Table 8:** Sutures group

Mesh	No	HR	aHR (95%-CI)
Bard Soft	61 (32.3%)	0.30	n/a
UltraPro	128 (67.7%)	1 (ref)	1 (ref)
Large/Light	498 (76.4%)	1 (ref)	1 (ref)
Small/Light	0	-	-
Large/Heavy	68 (10.4%)	1.28	5.20 (0.90–30.2)
Small/Heavy	86 (13.2%)	2.02	4.70 (0.47–46.6)

**Table 9:** Tacker fixation group

Mesh	No	HR	aHR (95%-CI)
Bard 3D	325 (1.7%)	0.24	0.31 (0.04–2.20)
Bard 3D Light	1,468 (7.8%)	0.52	***0.55 (0.35–0.87)***
Bard Soft	425 (2.3%)	0.80	1.49 (0.66–3.38)
Cousin	149 (0.8%)	1.06	1.39 (0.35–5.63)
Galmesh	1,775 (9.5%)	0.54	***0.39 (0.25–0.61)***
Galmesh Light	598 (3.2%)	0.74	0.69 (0.40–1.20)
Klas	106 (0.6%)	1.73	1.83 (0.80–4.15)
Optilene	1,937 (10.3%)	0.36	***0.28 (0.17–0.44)***
Optilene Elastic	244 (1.3%)	0.45	0.46 (0.17–1.25)
Optilene LP	207 (1.1%)	0.49	0.40 (0.13–1.25)
Parietene	2,674 (14.3%)	0.77	***0.62 (0.47–0.83)***
Parietex	2,161 (11.5%)	0.89	***0.70 (0.51–0.97)***
PhysioMesh	51 (0.3%)	1.97	0.79 (0.11–5.62)
Prolene	1,229 (6.6%)	0.93	***0.59 (0.37–0.93)***
SupraMesh	237 (1.3%)	0.64	1.26 (0.31–5.07)
SurgiPro	168 (0.9%)	1.37	1.89 (0.84–4.27)
TiMesh	75 (0.4%)	0.71	0.26 (0.04–1.89)
UltraPro	5,804 (31.0%)	1 (ref)	1 (ref)
UltraPro Adv	913 (4.9%)	0.53	***0.49 (0.32–0.75)***
Versatex Monofil	439 (2.3%)	0.81	0.62 (0.34–1.11)
VyPro	133 (0.7%)	1.44	1.17 (0.63–2.16)
Large/Light	14,486 (65.6%)	1 (ref)	1 (ref)
Small/Light	75 (0.3%)	0.86	0.34 (0.05–2.44)
Large/Heavy	3,327 (15.1%)	1.02	0.86 (0.66–1.12)
Small/Heavy	4,200 (19.0%)	0.90	0.77 (0.58–1.03)

There was no demonstrably significant difference in either crude HR or aHR when comparing meshes of various combinations of pore size and density for any fixation method, and the results was consistent with those listed in Table 4.

### Supplementary analyses of tack fixation

Of the 23,285 patients in the tack fixation group, a total of 7,305 received fixation with absorbable tacks and 11,223 with non-absorbable tacks, with a data insufficiency in 4,757 cases. In the absorbable group, 342 had a reoperation for recurrence (4.7%) compared to 532 (4.7%) in the non-absorbable group (*p = *0.854).

## Discussion

This analysis was carried out on a large, diverse, and unselected patient population over a 12-year study period. We found that despite being widely used, the tissue-penetrating fixation methods, and tack fixation in particular, carried a higher risk of reoperation for recurrence, regardless of mesh properties and absorbability of tacks. Meanwhile, the less extensively used non-penetrating methods, and to a lesser extent no fixation, had better results (Table 2).

The apparent superiority of non-penetrative fixation may owe to the fact that self-adhesive meshes and fixation with fibrin sealant enables fixation of the entire mesh, as opposed to tack fixation which omits fixation on the inferolateral edge of the mesh as to avoid damage to nerves and vessels.

Our results are consistent with other studies, although to our knowledge, this is the only large study that includes no fixation in an analysis of multiple fixation methods [11, 12, 14–16, 18–22, 24–31]. Previous studies have compared fixation to no fixation with no distinction in fixation methods [19, 22, 25], non-penetrative to penetrative fixation methods [10–12, 16, 21, 24, 36–42], or penetrative fixation to self-fixation [43] and have largely reported little or no differences. Several meta-analyses have been published; two compared tack fixation to no fixation [19, 25], four compared tack fixation to glue fixation [21, 23, 26, 28], two compared no fixation to all fixation [31], and one compared self-fixating mesh with conventional mesh [29], demonstrating non-superiority. One systematic review included trials comparing two methods of fixation or fixation to no fixation, but no trials compared multiple fixation methods to no fixation [44]. These studies have had smaller patient populations and shorter follow-up than the present study.

Our findings are in accordance with previous international recommendations of using non-penetrative fixation of mesh in an attempt to limit risk of chronic pain [45] and to use fixation on medial hernias [8]. More recent publications do not include this recommendation [8, 46, 47], only indicating a weak suggestion of atraumatic devices such as glue-fixation or fibrin sealant to reduce the risk of post-operative, acute pain. While concerns of pain development are not substantiated sufficiently to recommend non-penetrative mesh fixation (or no fixation for non-medial hernias), concerns of recurrence should be demonstrably weighted in these methods'favour.

We found little evidence of an independent impact of the interaction between choice of mesh and fixation method (Tables 5, 6, 7, 8, and 9). Of note, the Cousin mesh, which was among the meshes who had significantly lower hazard compared to the most widely used mesh (UltraPro) in the all-fixation method analysis (Table 3), faired significantly poorer in the no-fixation analysis (Table 6). There were numerous significant differences in tack fixation, though these matched the significant differences in the analysis including all fixation methods. Low-weight meshes (LWM) had no increased risk compared to high-weight meshes (HWM) regardless of pore size in the adjusted cox regression. This is largely consistent with previous studies: a large meta-analysis showed significant superiority of HWM when including non-fixated mesh repairs and no significant difference when analyzing only mesh fixation repairs [48].

The study is not without limitations. The apparent superiority of the no fixation group is vulnerable to bias, as less difficult cases with easier approachability and few complicating factors may lend themselves to fixationless mesh implantation. We also had no way of correcting for surgeon experience, and more experienced surgeons may gravitate towards certain fixation methods. The size of this potential effect is presumably minor, as procedures carried out by supervised residents was not associated with higher reoperation rates in this study. There was some data incompleteness on whether operations were supervised and perioperative finding of sliding hernia, but previous studies indicate that these factors are not associated with higher risk of recurrence [49, 50]. The Danish Hernia Database uses fingers width rather than a centimeter estimate to measure hernia defect size. This is to align with former European Hernia Society standards; these have however since been changed. There is at this point no indications of hernia defect size in centimeters or inches in the Danish Hernia Database.

We used reoperation as a surrogate for true recurrence, as the dataset did not include the latter. Studies show true recurrence rates to be almost 40% higher than reoperation rates [51]. Some patient groups are less likely to seek reoperation, and if these are distributed unevenly among the three analyzed groups, results may shift. In addition, the 5-year reoperation rate did not seem to linearly correlate to reoperation hazard. This suggests that when comparing factors in inguinal hernia repair related to recurrence, an even longer scale of time is needed to provide a more correct estimate of total life-time risk of reoperation. This is consistent with a previous study [52]. While the self-fixating mesh had the best results in this study, it also had the shortest mean follow-up (Fig. 2); more data on long-term recurrence risk is therefore needed before recommending self-fixation over, for example, glue fixation.

The lack of significant differences in the analyses of mesh model hazard for various fixation methods (Tables 5, 6, 7, 8, and 9) may be attributable to a type II error, when looking at a dataset with this many variables and mesh types, and comparably few light-weight/small pore meshes were included.

Per the data, we cannot say that there is such a thing as a right mesh for a given fixation method in TAPP, and either mesh or fixation method may be chosen by the quality of each factor independently.

Ideally, a randomized controlled study would be better fitted to test our study’s endpoints. However, randomizing a sufficient patient population to six different fixation methods (as have been compared in this study) and following them for a period of many years, in addition to accounting for mesh type, would be an arduous and unfeasible task. This, we feel, is the next best option.

There are numerous options available in inguinal hernia repair and even so specifically in TAPP repair. These findings demonstrate that hernia repair with glue fixation and self-fixating meshes carry low risk of reoperation, and may be a preferable approach to other fixation methods.

Choice of mesh also matters, and the mesh-related risk of recurrence is not necessarily dependent on easily categorized factors such as mesh weight or pore size. Recent guidelines recommend using HWM in TAPP [47]. This recommendation is based on the textile characteristics and vulnerability to bursting in tack fixation as well as higher risk of failure in direct and large defects [48]. We did not find that HWM meshes in particular improved results significantly, although some types of mesh had significantly superior results. Arguments for certain mesh-types based on their feasibility with tissue-penetrating fixation methods should be weighted by the apparent higher risk of reoperation carried by these methods.

In the end, several patient and operative related factors affect risk of recurrence. A plethora of different meshes exist and are continuously developed, making it difficult to attain strong data even on a large patient population. In this multifactorial environment, there is a need to be guided by strong evidence. Large, national, or even international, databases of mesh, surgeon, and patient characteristics as well as outcomes are needed to provide actionable data. Future efforts may advantageously make extensive use of such databases as an essential guide to mesh selection and quality assurance, to lastingly improve outcomes for this substantial patient group.

## Data Availability

Data can be made available upon reasonable requests.

## Declarations

This study meets the requirements of the STROBE statement checklist.
